# The Impact of Bilingualism on Cognitive Functioning in Older Adults

**DOI:** 10.1093/geroni/igaa057.947

**Published:** 2020-12-16

**Authors:** Hillary Rouse, Brent Small, John Schinka

**Affiliations:** 1 University of South Florida, Tampa, Florida, United States; 2 University of South Florida, tampa, Florida, United States

## Abstract

Research on bilingualism has found inconsistent results regarding its potential benefit on the cognitive abilities of older adults. The goal of the current study was to evaluate differences in cognition on a wide array of neuropsychological assessments between monolingual and bilingual cognitively healthy older adults who specifically speak only English and/or Spanish. The sample included cognitively intact older adults who were either monolingual (n=247) English speakers or bilingual (n=42) in English and Spanish. Performance was compared between groups from a battery of neuropsychological assessments that measured executive function, attention, short-term memory, and episodic memory. Compared to English and Spanish bilinguals, monolingual English speakers performed significantly better on a variety of tasks within the domains of executive function, attention, and short-term memory. No significant differences were found in favor of the bilinguals on any domain of cognitive performance. In the present study, we failed to observe a significant advantage for English and Spanish bilingual speakers on the cognitive performance of older adults when compared to monolingual English speakers. This study suggests that the bilingual advantage may not be as robust as originally reported, and the effects of bilingualism on cognition could be significantly impacted by the languages included in the study.

